# 2-(4-Chloro­phen­yl)-2-oxoethyl 2,4-di­fluoro­benzoate

**DOI:** 10.1107/S1600536811020630

**Published:** 2011-06-11

**Authors:** Hoong-Kun Fun, Suhana Arshad, B. Garudachari, Arun M. Isloor, M. N. Satyanarayan

**Affiliations:** a X-ray Crystallography Unit, School of Physics, Universiti Sains Malaysia, 11800 USM, Penang, Malaysia; b Organic Chemistry Division, Department of Chemistry, National Institute of Technology-Karnataka, Surathkal, Mangalore 575 025, India; c Department of Physics, National Institute of Technology-Karnataka, Surathkal, Mangalore 575 025, India

## Abstract

The asymmetric unit of title compound, C_15_H_9_ClF_2_O_3_, consists of two crystallographically independent mol­ecules. The dihedral angle between the two terminal benzene rings in one mol­ecule is 7.92 (14)°, while that in the other mol­ecule is 73.50 (16)°. In the crystal, mol­ecules are stacked into columns along the *b* axis by inter­molecular C—H⋯O hydrogen bonds. A π–π inter­action with a centroid-to-centroid distance of 3.747 (2) Å further stabilizes the crystal structure.

## Related literature

For background to and applications of phenacyl benzoates, see: Rather & Reid (1919 ▶); Sheehan & Umezaw (1973 ▶); Ruzicka *et al.* (2002 ▶); Litera *et al.* (2006 ▶); Huang *et al.* (1996 ▶); Gandhi *et al.* (1995 ▶). For reference bond-length values, see: Allen *et al.* (1987 ▶).
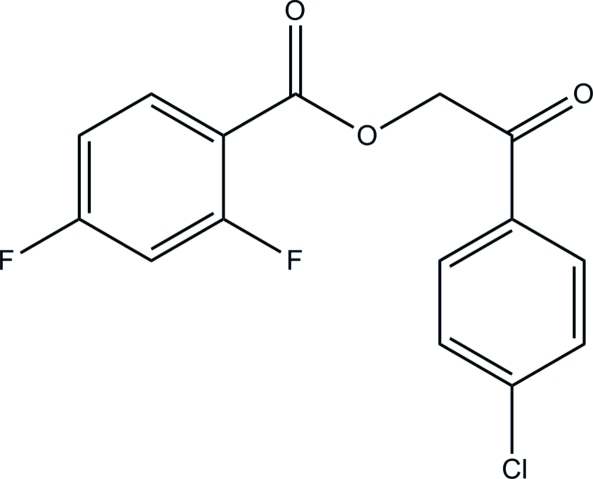


## Experimental

### 

#### Crystal data

C_15_H_9_ClF_2_O_3_
*M* *_r_* = 310.67
Monoclinic, 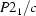

*a* = 16.0179 (17) Å
*b* = 7.9609 (8) Å
*c* = 24.0172 (18) Å
β = 115.939 (5)°
*V* = 2754.1 (5) Å^3^
*Z* = 8
Mo *K*α radiation
μ = 0.31 mm^−1^
*T* = 296 K
0.55 × 0.26 × 0.09 mm

#### Data collection

Bruker SMART APEXII DUO CCD area-detector diffractometer
Absorption correction: multi-scan (*SADABS*; Bruker, 2009 ▶) *T*_min_ = 0.850, *T*_max_ = 0.974
17424 measured reflections
6308 independent reflections
3353 reflections with *I* > 2σ(*I*)
*R*_int_ = 0.030

#### Refinement

*R*[*F* ^2^ > 2σ(*F* ^2^)] = 0.054
*wR*(*F* ^2^) = 0.206
*S* = 1.03
6308 reflections
379 parameters
H-atom parameters constrained
Δρ_max_ = 0.33 e Å^−3^
Δρ_min_ = −0.32 e Å^−3^

### 

Data collection: *APEX2* (Bruker, 2009 ▶); cell refinement: *SAINT* (Bruker, 2009 ▶); data reduction: *SAINT*; program(s) used to solve structure: *SHELXTL* (Sheldrick, 2008 ▶); program(s) used to refine structure: *SHELXTL*; molecular graphics: *SHELXTL*; software used to prepare material for publication: *SHELXTL* and *PLATON* (Spek, 2009 ▶).

## Supplementary Material

Crystal structure: contains datablock(s) global, I. DOI: 10.1107/S1600536811020630/is2722sup1.cif

Structure factors: contains datablock(s) I. DOI: 10.1107/S1600536811020630/is2722Isup2.hkl

Supplementary material file. DOI: 10.1107/S1600536811020630/is2722Isup3.cml

Additional supplementary materials:  crystallographic information; 3D view; checkCIF report

## Figures and Tables

**Table 1:** Hydrogen-bond geometry (Å, °)

*D*—H⋯*A*	*D*—H	H⋯*A*	*D*⋯*A*	*D*—H⋯*A*
C8*A*—H8*AB*⋯O3*B*	0.97	2.60	3.451 (4)	147
C8*A*—H8*AA*⋯O1*B*^i^	0.97	2.42	3.294 (3)	149
C5*B*—H5*BA*⋯O3*A*^ii^	0.93	2.50	3.376 (4)	158
C8*B*—H8*BB*⋯O3*A*^ii^	0.97	2.58	3.415 (3)	144
C14*B*—H14*B*⋯O1*A*^iii^	0.93	2.59	3.216 (5)	125

Symmetry codes: (i) 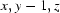; (ii) 
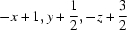
; (iii) 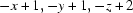.

**Fig. 1.**
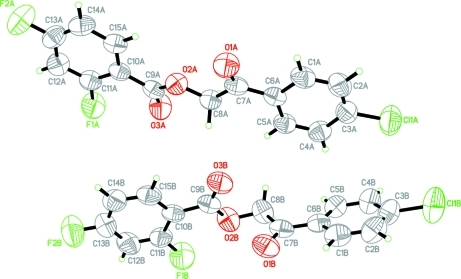
The molecular structure of the title compound, showing two independent molecules with atom labels with 50% probability displacement ellipsoids.

**Fig. 2.**
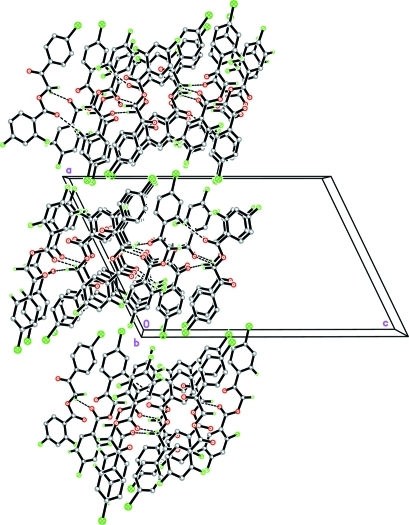
The crystal packing of the title compound. Dashed lines represent the hydrogen bonds.

## supplementary crystallographic information

### Comment

Phenacyl benzoates derivatives are very important in identification of organic acids (Rather & Reid, 1919), they undergo photolysis in neutral and mild conditions (Sheehan & Umezaw, 1973; Ruzicka *et al.*, 2002; Litera *et al.*, 2006). They find applications in the field of synthetic chemistry for the synthesis of oxazoles, imidazoles (Huang *et al.*, 1996), benzoxazepine (Gandhi *et al.*, 1995). We hereby report the crystal structure of 2-(4-chlorophenyl)-2-oxoethyl 2,4-difluorobenzoate of potential commercial importance.

The asymmetric unit of the title compound (Fig. 1), consists of two crystallographically independent molecules *A* and *B*. Both terminal phenyl rings (C1–C6 and C10–C15) in molecules *A* and *B* make dihedral angles of 7.92 (14) and 73.50 (16)° to each other, respectively. The bond lengths (Allen *et al.*, 1987) and angles are within normal ranges.

The crystal packing is shown in Fig. 2. The intermolecular C8A—H8AB···O3B hydrogen bond linked the molecule *A* with molecule *B* together. The molecules are linked into columns along the *b* axis by the intermolecular C8A—H8AA···O1B, C5B—H5BA···O3A, C8B—H8BB···O3A and C14B—H14B···O1A hydrogen bonds (Table 1). A π–π interaction further stabilizes the crystal structure [*Cg*1···*Cg*2^ii^ = 3.747 (2) Å; *Cg*1 and *Cg*2 are centroids of C1B–C6B and C10B–C15B benzene ring, respectively].

### Experimental

A mixture of 2,4-difluorobenzoic acid (1.0 g, 0.0063 mol) potassium carbonate (0.95 g, 0.0069 mol) and 2-bromo-1-(4-chlorophenyl)ethanone (1.41 g, 0.0063 mol) in dimethylformamide (10 ml) was stirred at room temperature for 2 h. On cooling, colourless needle-shaped crystals 2-(4-chlorophenyl)-2-oxoethyl 2,4-difluorobenzoate begin to separate. It was collected by filtration and recrystallized from ethanol. Yield: 1.65 g, 84.1%. M.p.: 376–377 K.

### Refinement

All H atoms were positioned geometrically (C—H = 0.93 or 0.97 Å) and refined using a riding model with *U*_iso_(H) = 1.2*U*_eq_(C). Four reflections, -6 4 4, -6 5 8, -1 1 1 and -1 4 10, were omitted.

### Crystal data

**Table:**

C_15_H_9_ClF_2_O_3_	*F*(000) = 1264
*M**_r_* = 310.67	*D*_x_ = 1.499 Mg m^−^^3^
Monoclinic, *P*2_1_/*c*	Mo *K*α radiation, λ = 0.71073 Å
Hall symbol: -P 2ybc	Cell parameters from 3882 reflections
*a* = 16.0179 (17) Å	θ = 2.6–22.5°
*b* = 7.9609 (8) Å	µ = 0.31 mm^−^^1^
*c* = 24.0172 (18) Å	*T* = 296 K
β = 115.939 (5)°	Needle, colourless
*V* = 2754.1 (5) Å^3^	0.55 × 0.26 × 0.09 mm
*Z* = 8	

### Data collection

**Table:**

Bruker SMART APEXII DUO CCD area-detector diffractometer	6308 independent reflections
Radiation source: fine-focus sealed tube	3353 reflections with *I* > 2σ(*I*)
graphite	*R*_int_ = 0.030
φ and ω scans	θ_max_ = 27.5°, θ_min_ = 1.8°
Absorption correction: multi-scan (*SADABS*; Bruker, 2009)	*h* = −20→19
*T*_min_ = 0.850, *T*_max_ = 0.974	*k* = −10→10
17424 measured reflections	*l* = −31→31

### Refinement

**Table:**

Refinement on *F*^2^	Primary atom site location: structure-invariant direct methods
Least-squares matrix: full	Secondary atom site location: difference Fourier map
*R*[*F*^2^ > 2σ(*F*^2^)] = 0.054	Hydrogen site location: inferred from neighbouring sites
*wR*(*F*^2^) = 0.206	H-atom parameters constrained
*S* = 1.03	*w* = 1/[σ^2^(*F*_o_^2^) + (0.114*P*)^2^ + 0.0306*P*] where *P* = (*F*_o_^2^ + 2*F*_c_^2^)/3
6308 reflections	(Δ/σ)_max_ = 0.001
379 parameters	Δρ_max_ = 0.33 e Å^−^^3^
0 restraints	Δρ_min_ = −0.32 e Å^−^^3^

### Special details

**Table:**

Geometry. All e.s.d.'s (except the e.s.d. in the dihedral angle between two l.s. planes) are estimated using the full covariance matrix. The cell e.s.d.'s are taken into account individually in the estimation of e.s.d.'s in distances, angles and torsion angles; correlations between e.s.d.'s in cell parameters are only used when they are defined by crystal symmetry. An approximate (isotropic) treatment of cell e.s.d.'s is used for estimating e.s.d.'s involving l.s. planes.
Refinement. Refinement of *F*^2^ against ALL reflections. The weighted *R*-factor *wR* and goodness of fit *S* are based on *F*^2^, conventional *R*-factors *R* are based on *F*, with *F* set to zero for negative *F*^2^. The threshold expression of *F*^2^ > σ(*F*^2^) is used only for calculating *R*-factors(gt) *etc*. and is not relevant to the choice of reflections for refinement. *R*-factors based on *F*^2^ are statistically about twice as large as those based on *F*, and *R*-factors based on ALL data will be even larger.

### Fractional atomic coordinates and isotropic or equivalent isotropic displacement parameters (Å^2^)

**Table:**

	*x*	*y*	*z*	*U*_iso_*/*U*_eq_	
Cl1A	0.01833 (6)	0.69373 (15)	0.67059 (4)	0.1116 (4)	
F1A	0.77697 (12)	0.3081 (3)	1.00087 (8)	0.1006 (6)	
F2A	0.80582 (16)	−0.0433 (3)	1.16292 (10)	0.1139 (7)	
O1A	0.32955 (14)	0.3728 (3)	0.93888 (9)	0.0827 (6)	
O2A	0.49808 (12)	0.3539 (2)	0.95029 (8)	0.0654 (5)	
O3A	0.60473 (13)	0.3792 (3)	0.91508 (8)	0.0869 (7)	
C1A	0.1715 (2)	0.4728 (4)	0.83169 (14)	0.0734 (8)	
H1AA	0.1615	0.4076	0.8603	0.088*	
C2A	0.0965 (2)	0.5297 (4)	0.77968 (15)	0.0828 (9)	
H2AA	0.0364	0.5010	0.7727	0.099*	
C3A	0.11135 (19)	0.6286 (4)	0.73861 (13)	0.0718 (7)	
C4A	0.1996 (2)	0.6748 (4)	0.74836 (13)	0.0710 (7)	
H4AA	0.2086	0.7461	0.7208	0.085*	
C5A	0.27448 (19)	0.6134 (4)	0.79980 (12)	0.0661 (7)	
H5AA	0.3343	0.6413	0.8061	0.079*	
C6A	0.26202 (17)	0.5109 (3)	0.84226 (11)	0.0570 (6)	
C7A	0.34074 (18)	0.4379 (3)	0.89723 (11)	0.0589 (6)	
C8A	0.43456 (17)	0.4445 (3)	0.89777 (11)	0.0608 (6)	
H8AA	0.4319	0.3949	0.8601	0.073*	
H8AB	0.4547	0.5603	0.8999	0.073*	
C9A	0.58203 (18)	0.3275 (3)	0.95319 (11)	0.0590 (6)	
C10A	0.64078 (17)	0.2283 (3)	1.00863 (11)	0.0573 (6)	
C11A	0.73636 (19)	0.2215 (4)	1.03031 (12)	0.0667 (7)	
C12A	0.7933 (2)	0.1312 (4)	1.08180 (13)	0.0754 (8)	
H12A	0.8572	0.1285	1.0954	0.091*	
C13A	0.7515 (2)	0.0455 (4)	1.11226 (13)	0.0760 (8)	
C14A	0.6584 (2)	0.0462 (4)	1.09386 (13)	0.0758 (8)	
H14A	0.6325	−0.0152	1.1154	0.091*	
C15A	0.6027 (2)	0.1400 (3)	1.04240 (12)	0.0649 (7)	
H15A	0.5390	0.1444	1.0301	0.078*	
Cl1B	−0.01753 (7)	1.09979 (18)	0.57396 (5)	0.1307 (5)	
F1B	0.70241 (15)	1.0849 (3)	0.83037 (10)	0.1280 (9)	
F2B	0.92324 (16)	0.8868 (4)	1.01835 (10)	0.1338 (8)	
O1B	0.41561 (18)	1.1476 (3)	0.79738 (10)	0.1000 (7)	
O2B	0.54117 (14)	0.9566 (3)	0.78473 (8)	0.0834 (6)	
O3B	0.49789 (16)	0.8028 (3)	0.84487 (11)	0.1036 (8)	
C1B	0.2248 (3)	1.1648 (4)	0.71976 (14)	0.0828 (9)	
H1BA	0.2467	1.2199	0.7577	0.099*	
C2B	0.1322 (3)	1.1733 (5)	0.67968 (16)	0.0935 (10)	
H2BA	0.0915	1.2341	0.6902	0.112*	
C3B	0.1000 (2)	1.0906 (4)	0.62378 (13)	0.0838 (9)	
C4B	0.1590 (2)	1.0031 (4)	0.60703 (12)	0.0816 (9)	
H4BA	0.1365	0.9495	0.5688	0.098*	
C5B	0.2515 (2)	0.9953 (4)	0.64709 (11)	0.0717 (7)	
H5BA	0.2916	0.9357	0.6358	0.086*	
C6B	0.2865 (2)	1.0750 (3)	0.70449 (11)	0.0644 (7)	
C7B	0.3848 (2)	1.0667 (3)	0.74964 (12)	0.0696 (7)	
C8B	0.4479 (2)	0.9489 (5)	0.73696 (12)	0.0805 (9)	
H8BA	0.4248	0.8350	0.7339	0.097*	
H8BB	0.4475	0.9778	0.6976	0.097*	
C9B	0.5572 (2)	0.8795 (4)	0.83760 (13)	0.0702 (7)	
C10B	0.65445 (19)	0.8922 (3)	0.88491 (12)	0.0629 (7)	
C11B	0.7239 (2)	0.9844 (4)	0.88009 (13)	0.0757 (8)	
C12B	0.8133 (2)	0.9838 (5)	0.92315 (15)	0.0882 (9)	
H12B	0.8582	1.0466	0.9179	0.106*	
C13B	0.8352 (2)	0.8877 (5)	0.97465 (14)	0.0862 (9)	
C14B	0.7698 (3)	0.7944 (4)	0.98378 (14)	0.0864 (9)	
H14B	0.7858	0.7300	1.0193	0.104*	
C15B	0.6810 (2)	0.8001 (4)	0.93878 (13)	0.0757 (8)	
H15B	0.6359	0.7390	0.9445	0.091*	

### Atomic displacement parameters (Å^2^)

**Table:**

	*U*^11^	*U*^22^	*U*^33^	*U*^12^	*U*^13^	*U*^23^
Cl1A	0.0784 (6)	0.1292 (9)	0.1112 (7)	0.0192 (5)	0.0266 (5)	0.0172 (6)
F1A	0.0685 (11)	0.1358 (18)	0.1080 (13)	−0.0061 (10)	0.0484 (10)	0.0250 (11)
F2A	0.1271 (17)	0.1155 (17)	0.1067 (13)	0.0402 (13)	0.0580 (13)	0.0393 (12)
O1A	0.0813 (13)	0.0993 (16)	0.0837 (12)	−0.0051 (11)	0.0511 (11)	0.0162 (11)
O2A	0.0666 (11)	0.0714 (12)	0.0692 (10)	0.0014 (9)	0.0398 (9)	0.0027 (9)
O3A	0.0691 (12)	0.130 (2)	0.0746 (11)	−0.0026 (11)	0.0432 (10)	0.0174 (11)
C1A	0.0694 (18)	0.0748 (19)	0.0922 (18)	−0.0099 (15)	0.0504 (16)	0.0013 (15)
C2A	0.0612 (17)	0.091 (2)	0.107 (2)	−0.0066 (15)	0.0467 (17)	0.0037 (18)
C3A	0.0655 (17)	0.0686 (18)	0.0827 (17)	0.0034 (14)	0.0337 (14)	−0.0080 (14)
C4A	0.0788 (19)	0.0675 (18)	0.0772 (16)	0.0013 (14)	0.0437 (15)	0.0051 (14)
C5A	0.0656 (16)	0.0652 (17)	0.0790 (16)	−0.0064 (13)	0.0420 (14)	−0.0023 (13)
C6A	0.0631 (15)	0.0524 (14)	0.0665 (13)	−0.0075 (11)	0.0386 (12)	−0.0110 (11)
C7A	0.0722 (17)	0.0505 (14)	0.0670 (14)	−0.0098 (12)	0.0426 (13)	−0.0107 (12)
C8A	0.0652 (16)	0.0622 (16)	0.0629 (13)	−0.0041 (12)	0.0352 (12)	−0.0022 (12)
C9A	0.0619 (15)	0.0620 (16)	0.0619 (13)	−0.0168 (12)	0.0352 (12)	−0.0134 (12)
C10A	0.0647 (15)	0.0523 (15)	0.0632 (13)	−0.0099 (12)	0.0356 (12)	−0.0121 (11)
C11A	0.0684 (17)	0.0688 (18)	0.0739 (15)	−0.0073 (14)	0.0414 (14)	−0.0037 (13)
C12A	0.0723 (18)	0.078 (2)	0.0825 (17)	0.0069 (15)	0.0396 (15)	−0.0008 (15)
C13A	0.096 (2)	0.0638 (18)	0.0739 (16)	0.0151 (16)	0.0419 (16)	0.0045 (14)
C14A	0.099 (2)	0.0632 (18)	0.0848 (18)	−0.0055 (16)	0.0580 (18)	0.0019 (15)
C15A	0.0722 (17)	0.0585 (16)	0.0745 (15)	−0.0096 (13)	0.0416 (14)	−0.0075 (13)
Cl1B	0.0942 (7)	0.1856 (13)	0.1040 (7)	0.0478 (7)	0.0356 (6)	0.0041 (7)
F1B	0.1063 (16)	0.154 (2)	0.1211 (15)	−0.0321 (14)	0.0474 (13)	0.0536 (14)
F2B	0.0901 (15)	0.181 (2)	0.1081 (15)	−0.0141 (15)	0.0231 (13)	0.0130 (15)
O1B	0.1165 (19)	0.0871 (16)	0.0938 (15)	−0.0155 (13)	0.0435 (14)	−0.0334 (12)
O2B	0.0794 (14)	0.1104 (17)	0.0709 (11)	−0.0151 (12)	0.0426 (11)	−0.0034 (11)
O3B	0.0879 (16)	0.0978 (17)	0.1253 (18)	−0.0294 (13)	0.0468 (14)	0.0205 (14)
C1B	0.109 (3)	0.075 (2)	0.0801 (18)	0.0001 (17)	0.0553 (19)	−0.0151 (15)
C2B	0.106 (3)	0.095 (3)	0.096 (2)	0.026 (2)	0.059 (2)	−0.0040 (19)
C3B	0.085 (2)	0.097 (2)	0.0764 (17)	0.0228 (17)	0.0423 (16)	0.0098 (16)
C4B	0.087 (2)	0.102 (2)	0.0608 (15)	0.0152 (17)	0.0365 (16)	−0.0038 (15)
C5B	0.085 (2)	0.078 (2)	0.0655 (15)	0.0082 (15)	0.0451 (15)	−0.0012 (13)
C6B	0.0864 (19)	0.0554 (16)	0.0664 (14)	−0.0021 (13)	0.0472 (14)	0.0018 (12)
C7B	0.093 (2)	0.0588 (17)	0.0702 (15)	−0.0190 (14)	0.0481 (16)	−0.0099 (13)
C8B	0.077 (2)	0.103 (2)	0.0665 (15)	−0.0066 (16)	0.0357 (15)	−0.0138 (15)
C9B	0.0790 (19)	0.0623 (17)	0.0821 (17)	−0.0141 (15)	0.0470 (15)	−0.0104 (14)
C10B	0.0774 (18)	0.0541 (16)	0.0727 (15)	−0.0107 (13)	0.0473 (14)	−0.0084 (12)
C11B	0.089 (2)	0.0713 (19)	0.0778 (17)	−0.0086 (16)	0.0466 (17)	0.0091 (15)
C12B	0.080 (2)	0.100 (3)	0.094 (2)	−0.0243 (18)	0.0477 (18)	−0.0006 (19)
C13B	0.075 (2)	0.103 (3)	0.0771 (18)	−0.0093 (18)	0.0295 (16)	−0.0089 (17)
C14B	0.107 (3)	0.086 (2)	0.0711 (17)	−0.0115 (19)	0.0433 (18)	0.0055 (16)
C15B	0.095 (2)	0.0682 (19)	0.0792 (17)	−0.0194 (15)	0.0523 (17)	−0.0038 (14)

### Geometric parameters (Å, °)

**Table:**

Cl1A—C3A	1.740 (3)	Cl1B—C3B	1.735 (3)
F1A—C11A	1.342 (3)	F1B—C11B	1.351 (3)
F2A—C13A	1.346 (3)	F2B—C13B	1.340 (4)
O1A—C7A	1.207 (3)	O1B—C7B	1.216 (3)
O2A—C9A	1.332 (3)	O2B—C9B	1.330 (3)
O2A—C8A	1.423 (3)	O2B—C8B	1.432 (3)
O3A—C9A	1.196 (3)	O3B—C9B	1.204 (3)
C1A—C2A	1.377 (4)	C1B—C2B	1.372 (5)
C1A—C6A	1.392 (4)	C1B—C6B	1.393 (4)
C1A—H1AA	0.9300	C1B—H1BA	0.9300
C2A—C3A	1.362 (4)	C2B—C3B	1.377 (5)
C2A—H2AA	0.9300	C2B—H2BA	0.9300
C3A—C4A	1.378 (4)	C3B—C4B	1.368 (4)
C4A—C5A	1.381 (4)	C4B—C5B	1.370 (4)
C4A—H4AA	0.9300	C4B—H4BA	0.9300
C5A—C6A	1.387 (3)	C5B—C6B	1.393 (3)
C5A—H5AA	0.9300	C5B—H5BA	0.9300
C6A—C7A	1.488 (4)	C6B—C7B	1.472 (4)
C7A—C8A	1.498 (3)	C7B—C8B	1.504 (4)
C8A—H8AA	0.9700	C8B—H8BA	0.9700
C8A—H8AB	0.9700	C8B—H8BB	0.9700
C9A—C10A	1.479 (4)	C9B—C10B	1.477 (4)
C10A—C11A	1.386 (4)	C10B—C11B	1.380 (4)
C10A—C15A	1.399 (3)	C10B—C15B	1.382 (4)
C11A—C12A	1.375 (4)	C11B—C12B	1.350 (4)
C12A—C13A	1.370 (4)	C12B—C13B	1.363 (4)
C12A—H12A	0.9300	C12B—H12B	0.9300
C13A—C14A	1.357 (4)	C13B—C14B	1.378 (5)
C14A—C15A	1.384 (4)	C14B—C15B	1.360 (4)
C14A—H14A	0.9300	C14B—H14B	0.9300
C15A—H15A	0.9300	C15B—H15B	0.9300
			
C9A—O2A—C8A	116.13 (18)	C9B—O2B—C8B	116.3 (2)
C2A—C1A—C6A	121.3 (3)	C2B—C1B—C6B	121.0 (3)
C2A—C1A—H1AA	119.3	C2B—C1B—H1BA	119.5
C6A—C1A—H1AA	119.3	C6B—C1B—H1BA	119.5
C3A—C2A—C1A	119.2 (3)	C1B—C2B—C3B	119.3 (3)
C3A—C2A—H2AA	120.4	C1B—C2B—H2BA	120.4
C1A—C2A—H2AA	120.4	C3B—C2B—H2BA	120.4
C2A—C3A—C4A	121.5 (3)	C4B—C3B—C2B	121.2 (3)
C2A—C3A—Cl1A	120.2 (2)	C4B—C3B—Cl1B	120.0 (2)
C4A—C3A—Cl1A	118.3 (2)	C2B—C3B—Cl1B	118.8 (2)
C3A—C4A—C5A	118.8 (3)	C3B—C4B—C5B	119.4 (3)
C3A—C4A—H4AA	120.6	C3B—C4B—H4BA	120.3
C5A—C4A—H4AA	120.6	C5B—C4B—H4BA	120.3
C4A—C5A—C6A	121.2 (2)	C4B—C5B—C6B	121.2 (3)
C4A—C5A—H5AA	119.4	C4B—C5B—H5BA	119.4
C6A—C5A—H5AA	119.4	C6B—C5B—H5BA	119.4
C5A—C6A—C1A	117.9 (3)	C1B—C6B—C5B	118.0 (3)
C5A—C6A—C7A	122.9 (2)	C1B—C6B—C7B	118.9 (2)
C1A—C6A—C7A	119.2 (2)	C5B—C6B—C7B	123.1 (2)
O1A—C7A—C6A	121.8 (2)	O1B—C7B—C6B	122.2 (3)
O1A—C7A—C8A	121.3 (2)	O1B—C7B—C8B	119.3 (3)
C6A—C7A—C8A	116.85 (19)	C6B—C7B—C8B	118.4 (2)
O2A—C8A—C7A	108.40 (19)	O2B—C8B—C7B	111.6 (2)
O2A—C8A—H8AA	110.0	O2B—C8B—H8BA	109.3
C7A—C8A—H8AA	110.0	C7B—C8B—H8BA	109.3
O2A—C8A—H8AB	110.0	O2B—C8B—H8BB	109.3
C7A—C8A—H8AB	110.0	C7B—C8B—H8BB	109.3
H8AA—C8A—H8AB	108.4	H8BA—C8B—H8BB	108.0
O3A—C9A—O2A	122.9 (2)	O3B—C9B—O2B	122.5 (3)
O3A—C9A—C10A	125.9 (2)	O3B—C9B—C10B	123.8 (3)
O2A—C9A—C10A	111.19 (19)	O2B—C9B—C10B	113.6 (2)
C11A—C10A—C15A	116.9 (2)	C11B—C10B—C15B	115.6 (3)
C11A—C10A—C9A	121.5 (2)	C11B—C10B—C9B	126.2 (2)
C15A—C10A—C9A	121.5 (2)	C15B—C10B—C9B	118.2 (2)
F1A—C11A—C12A	117.3 (2)	C12B—C11B—F1B	117.0 (3)
F1A—C11A—C10A	119.5 (2)	C12B—C11B—C10B	123.9 (3)
C12A—C11A—C10A	123.2 (2)	F1B—C11B—C10B	119.1 (3)
C13A—C12A—C11A	117.0 (3)	C11B—C12B—C13B	117.6 (3)
C13A—C12A—H12A	121.5	C11B—C12B—H12B	121.2
C11A—C12A—H12A	121.5	C13B—C12B—H12B	121.2
F2A—C13A—C14A	118.7 (3)	F2B—C13B—C12B	118.6 (3)
F2A—C13A—C12A	118.1 (3)	F2B—C13B—C14B	119.0 (3)
C14A—C13A—C12A	123.2 (3)	C12B—C13B—C14B	122.3 (3)
C13A—C14A—C15A	118.7 (3)	C15B—C14B—C13B	117.3 (3)
C13A—C14A—H14A	120.7	C15B—C14B—H14B	121.3
C15A—C14A—H14A	120.7	C13B—C14B—H14B	121.3
C14A—C15A—C10A	121.0 (3)	C14B—C15B—C10B	123.3 (3)
C14A—C15A—H15A	119.5	C14B—C15B—H15B	118.4
C10A—C15A—H15A	119.5	C10B—C15B—H15B	118.4
			
C6A—C1A—C2A—C3A	−1.6 (5)	C6B—C1B—C2B—C3B	0.4 (5)
C1A—C2A—C3A—C4A	−0.9 (5)	C1B—C2B—C3B—C4B	−1.3 (5)
C1A—C2A—C3A—Cl1A	176.8 (2)	C1B—C2B—C3B—Cl1B	179.1 (3)
C2A—C3A—C4A—C5A	2.6 (4)	C2B—C3B—C4B—C5B	1.1 (5)
Cl1A—C3A—C4A—C5A	−175.1 (2)	Cl1B—C3B—C4B—C5B	−179.3 (3)
C3A—C4A—C5A—C6A	−1.8 (4)	C3B—C4B—C5B—C6B	−0.1 (5)
C4A—C5A—C6A—C1A	−0.5 (4)	C2B—C1B—C6B—C5B	0.5 (4)
C4A—C5A—C6A—C7A	178.1 (2)	C2B—C1B—C6B—C7B	−178.6 (3)
C2A—C1A—C6A—C5A	2.3 (4)	C4B—C5B—C6B—C1B	−0.7 (4)
C2A—C1A—C6A—C7A	−176.4 (3)	C4B—C5B—C6B—C7B	178.4 (3)
C5A—C6A—C7A—O1A	167.5 (2)	C1B—C6B—C7B—O1B	−6.7 (4)
C1A—C6A—C7A—O1A	−13.9 (4)	C5B—C6B—C7B—O1B	174.1 (3)
C5A—C6A—C7A—C8A	−14.2 (3)	C1B—C6B—C7B—C8B	170.9 (3)
C1A—C6A—C7A—C8A	164.4 (2)	C5B—C6B—C7B—C8B	−8.2 (4)
C9A—O2A—C8A—C7A	171.1 (2)	C9B—O2B—C8B—C7B	75.9 (3)
O1A—C7A—C8A—O2A	4.4 (3)	O1B—C7B—C8B—O2B	−2.8 (4)
C6A—C7A—C8A—O2A	−173.96 (19)	C6B—C7B—C8B—O2B	179.4 (2)
C8A—O2A—C9A—O3A	1.6 (4)	C8B—O2B—C9B—O3B	2.2 (4)
C8A—O2A—C9A—C10A	−178.4 (2)	C8B—O2B—C9B—C10B	−179.9 (2)
O3A—C9A—C10A—C11A	16.9 (4)	O3B—C9B—C10B—C11B	−176.8 (3)
O2A—C9A—C10A—C11A	−163.1 (2)	O2B—C9B—C10B—C11B	5.3 (4)
O3A—C9A—C10A—C15A	−164.6 (3)	O3B—C9B—C10B—C15B	5.4 (4)
O2A—C9A—C10A—C15A	15.4 (3)	O2B—C9B—C10B—C15B	−172.5 (2)
C15A—C10A—C11A—F1A	−177.9 (2)	C15B—C10B—C11B—C12B	2.1 (4)
C9A—C10A—C11A—F1A	0.7 (4)	C9B—C10B—C11B—C12B	−175.8 (3)
C15A—C10A—C11A—C12A	1.1 (4)	C15B—C10B—C11B—F1B	−176.4 (3)
C9A—C10A—C11A—C12A	179.7 (2)	C9B—C10B—C11B—F1B	5.7 (4)
F1A—C11A—C12A—C13A	178.9 (3)	F1B—C11B—C12B—C13B	177.6 (3)
C10A—C11A—C12A—C13A	−0.2 (4)	C10B—C11B—C12B—C13B	−0.9 (5)
C11A—C12A—C13A—F2A	−179.8 (2)	C11B—C12B—C13B—F2B	−179.2 (3)
C11A—C12A—C13A—C14A	0.1 (4)	C11B—C12B—C13B—C14B	−0.5 (5)
F2A—C13A—C14A—C15A	178.8 (2)	F2B—C13B—C14B—C15B	179.2 (3)
C12A—C13A—C14A—C15A	−1.1 (4)	C12B—C13B—C14B—C15B	0.5 (5)
C13A—C14A—C15A—C10A	2.1 (4)	C13B—C14B—C15B—C10B	0.9 (5)
C11A—C10A—C15A—C14A	−2.1 (4)	C11B—C10B—C15B—C14B	−2.1 (4)
C9A—C10A—C15A—C14A	179.4 (2)	C9B—C10B—C15B—C14B	176.0 (3)

### Hydrogen-bond geometry (Å, °)

**Table:**

*D*—H···*A*	*D*—H	H···*A*	*D*···*A*	*D*—H···*A*
C8A—H8AB···O3B	0.97	2.60	3.451 (4)	147.
C8A—H8AA···O1B^i^	0.97	2.42	3.294 (3)	149.
C5B—H5BA···O3A^ii^	0.93	2.50	3.376 (4)	158.
C8B—H8BB···O3A^ii^	0.97	2.58	3.415 (3)	144.
C14B—H14B···O1A^iii^	0.93	2.59	3.216 (5)	125.

Symmetry codes: (i) *x*, *y*−1, *z*; (ii) −*x*+1, *y*+1/2, −*z*+3/2; (iii) −*x*+1, −*y*+1, −*z*+2.
